# The effect of different endotracheal tube cuff pressure monitoring systems on postoperative sore throat in patients undergoing tracheal intubation: a randomized clinical trial

**DOI:** 10.1186/s12871-024-02499-5

**Published:** 2024-03-25

**Authors:** Guangli Zhu, Xuan Wang, Xinyu Cao, Chongya Yang, Bin Wang, Yang Ang, Manlin Duan

**Affiliations:** 1 College of Anesthesiology, Xuzhou Medical University, Xuzhou, Jiangsu 221004 China; 2 Department of Anesthesiology, Affiliated Jinling Hospital, Medical School, Nanjing University, Nanjing, Jiangsu Province 210002 China; 3 Department of Anesthesiology, Nanjing BenQ Medical Center, The Affiliated BenQ Hospital of Nanjing Medical University, Nanjing, Jiangsu Province 210019 China; 4 Women’s Hospital of Nanjing Medical University, Nanjing, Jiangsu 210004 China; 5 Department of Anesthesiology, Jinling College affiliated to Nanjing Medical University, Nanjing, Jiangsu 210002 China

**Keywords:** Postoperative sore throat, Endotracheal tube cuff pressure, Gauge, Automated cuff controller

## Abstract

**Background:**

Postoperative sore throat (POST) is an unpleasant outcome that can occur as a result of tracheal intubation in adults. Increased pressure from the endotracheal tube (ETT) cuff often leads to local mucosal injury, resulting in sore throat. The purpose of this study was to compare the effect of two different ETT cuff pressure monitoring systems vs. no cuff pressure monitoring on the incidence and severity of POST in adults.

**Methods:**

One hundred and fourteen ASA I-III patients of either gender, aged 18–65 years, and undergoing surgery requiring endotracheal intubation were included in this study. Patients were randomized into three groups: control (C), cuff pressure gauge (G), and automated cuff controller (A). The ETT cuff pressure was not monitored intraoperatively in group C but was monitored using a cuff pressure gauge and an automated cuff controller in groups G and A, respectively. Postoperatively, patients were assessed at 2, 24, and 48 h for the presence and severity of POST, hoarseness and cough.

**Results:**

One hundred and eleven patients completed the study. POST occurred in 40.5% of the patients in group G (*n* = 37) (*p* = 0.013) and 23.7% of the patients in group A (*n* = 38) (*p* < 0.001) within 48 h after surgery, compared to 69.4% in group C (*n* = 36). There were no significant differences in hoarseness, coughing, and dysphagia across the groups at any time. When comparing groups A and C, individuals in group A exhibited a lower occurrence of significant (grade ≥ 2) POST and hoarseness (10.5% vs. 41.7%, *p* = 0.002; 26.3% vs. 58.3%, *p* = 0.005). The incidence of significant cough and dysphagia did not differ substantially across the patient groups within 48 h after surgery. POST scores in group A at 2, 24 h postoperatively were both 0 (0–0), which was significantly lower than those in group C (1 (0–2) at 2 h, *p* < 0.001 ; 1 (0–1) at 24 h, *p* = 0.001). POST in group G at 2 h postoperatively was graded as 0 (0–1.5) which was milder than group C (*P* = 0.024). The severity of hoarseness in group A with scores of 0 (0–2) was superior to that in group C (2 (0–2), *p* = 0.006) at 2 h postoperatively.

**Conclusions:**

In conclusion, the findings of this study indicated that the occurrence of POST can be reduced by using either the cuff pressure gauge approach or the automated cuff controller method. The automated cuff controller monitoring can potentially decrease the severity of POST and hoarseness.

**Trial registration:**

Chinese Clinical Trial Registry, identifier: ChiCTR2100054089, Date: 08/12/2021.

**Supplementary Information:**

The online version contains supplementary material available at 10.1186/s12871-024-02499-5.

## Background

Postoperative sore throat (POST) is an uncomfortable condition that occurs in adults following endotracheal intubation, with the incidence of 30‒70% [1, 2]. The underlying reason is the mechanical stimulation of the airway mucosa caused by endotracheal intubation [3]. Hence, factors such as the technique used for endotracheal intubation, the size of the tube, and the pressures exerted by the cuffs all have significant effects [4–6].

The control of endotracheal tube (ETT) cuff pressure during surgery is an integral aspect of anesthesia. The prevention of regurgitant aspiration and airway damage is possible by keeping the ETT cuff pressure at 25–30 cmH_2_O (1 cmH_2_O = 0.098 KPa) [7]. If the cuff pressure on an endotracheal tube (ETT) is more than 30 cmH_2_O, local tracheal mucosal perfusion is greatly reduced, increasing the risk of postoperative airway problems, including POST, hoarseness, and dysphagia [8, 9]. Based on a multicenter survey conducted in Nigeria, it was shown that only 31.1% of anesthesia and intensive care personnel were aware of the issue of accurate ETT cuff pressure, and a staggering 97% had never utilized a cuff pressure monitor [10]. Therefore, monitoring the pressure within the cuff of the ETT throughout the operation is crucial for avoiding airway damage.

A cuff pressure gauge was often employed for such purposes in earlier study [11, 12]. This strategy, however, is insensitive and inefficient. The introduction of an automated cuff controller has enabled real-time monitoring of cuff pressure and automatic inflation or deflation as needed [13–15]. However, current study on this device has mainly focused on its use in the postoperative intensive care unit rather than the operating theater [16, 17]. The effects of automated cuff controllers on postoperative airway discomfort such as sore throat have not been thoroughly explored.

The objective of this study was to determine if automated cuff controllers, as opposed to cuff pressure gauges, caused fewer and lesser POST and airway issues. The primary outcome was the frequency of POST in the 48 h following surgery. The incidence and severity of postoperative hoarseness, cough, and dysphagia were also measured as secondary outcomes, along with the incidence and severity of POST at 2, 24, and 48 h postoperatively. We hypothesized that the use of an automated cuff controller or a cuff pressure gauge can lower the frequency of POST.

## Methods

This randomized controlled study followed the CONSORT reporting guidelines [18] and was carried out at Jinling Hospital, Jinling School of Clinical Medicine, Nanjing Medical University. Written informed permission was obtained from all participants following approval from the hospital’s Study Ethics Committee on March 18, 2022 (Ethical Approval Number: 2022DZKY-024-01 Nanjing, China). Information on the trial is available in the Chinese Clinical Trial Registry (Registration No. ChiCTR2100054089, date: August 12, 2021).

The participants included adults (of either sex) between the ages of 18 and 65 years, in ASA physical status I, II, or III, and scheduled to have elective procedures under general endotracheal anesthesia. Patients with a body mass index below 19 kg/m^− 2^ or over 30 kg/m^− 2^, existing sore throat, hoarseness, cough, bleeding in the laryngeal mucosa, asthma, chronic obstructive pulmonary disease, smoking, difficult airway, respiratory tract infection during the past two weeks, who had undergone insertion of a nasogastric tube, who had psychiatric disorders, or had undergone oral and ENT surgery were excluded from the trial.

The patients were enrolled one day before the operation and randomly divided into groups at a 1:1:1 ratio using a computer-generated random number table by the designer. The anesthesiologist, who supervised procedures such as intubation and cuff monitoring, had access to the opaque envelopes with the allocation numbers. Two separate assessors examined the outcome measures after surgery. Telephone follow-up was used for the postoperative follow-up in cases where the patients were discharged early. The patients, data information analysts, and the two outcome assessors were not informed of the trial’s intervention.

**Fig. 1:**
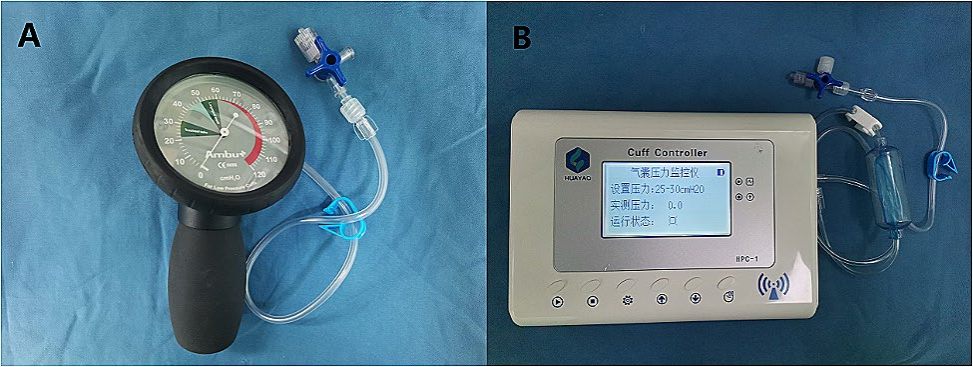
(**A**) shows the cuff pressure gauge (Ambu*R, Germany). This measures the instantaneous cuff pressure by connecting the cuff through a three-top. (**B**) shows the automated cuff controller (HPC-1, Wuxi Huayao Biotechnology Co., Ltd). This can continuously inflate and deflate to maintain the cuff pressure in the set safety range

The ETT cuffs of the patients in each group were inflated using a 10-ml syringe and the anesthesiologist’s usual method of pilot balloon palpation. Cuff pressure was not recorded in the control (C) group. A cuff pressure gauge (Ambu*R, Germany) (Fig. 1A) was used to check the ETT cuff pressure intraoperatively every hour in the cuff gauge (G) group, where it was maintained at 25–30 cmH_2_O throughout the procedure. The ETT cuff pressure was maintained constant at 25–30 cmH_2_O using an automated cuff controller (HPC-1, Wuxi Huayao Biotechnology Co., Ltd) in the automated (A) cuff controller group (Fig. 1B). In addition, the initial cuff pressure was also recorded when inflating the cuff according to the pilot ballon palpation in group G and A. Participants with a leaking ETT cuff were re-intubated and subsequently excluded from the study.

Sufentanil (0.4 µg.kg^− 1^), rocuronium (0.6 mg.kg^− 1^), and ciprofol (0.4 mg.kg^− 1^) were used to induce anesthesia. Visual laryngoscopy was used to guide the placement of an endotracheal tube (7.5 or 8.0 mm in males and 7.0 or 7.5 mm in females) reinforced with low-pressure, high-volume cuff (cuffed, Hisern Medical, Zhejiang, China) by an anesthesiologist with at least five years of clinical experience. Remifentanil (0.05-2.0 µg.kg^− 1^.min^− 1^), propofol (4.0–8.0 mg.kg^− 1^.h^− 1^), and cisatracurium (0.05 mg.kg^− 1^ every 30 min) were the drugs of choice for continuous general anesthesia induction and maintenance. The ideal body weight of the patient was used to determine the tidal volume (6*‒*8 ml.kg^− 1^), the respiratory rate (10*‒*13 breaths/min) and the peak airway pressure (25 mmHg). Mechanical ventilation was set to volume control. The patient was transferred to the post-anesthesia care unit (PACU) for postoperative resuscitation. Once the patient began breathing on their own and could follow directions by shaking hands, the endotracheal tube was removed by full deflation of the cuff after gentle suction of the oral cavity at a negative pressure of 50 cmH_2_O [19].

After surgery, patients were monitored at 2, 24, and 48 h to assess the prevalence and severity of POST, hoarseness, and cough. The occurrence and intensity of dysphagia were also recorded at 24 and 48 h. POST has been defined as an unpleasant feeling of discomfort or irritation experienced while at rest or swallowing. As previously stated, there were four categories delineating the severity of the concerns. An absence of POST was designated as grade 0, while grade 1 was milder than the normal cold, grade 2 almost as bad as the common cold, and grade 3 was very bad [20]. Those without a cough were assigned a rating of 0, those with a light or infrequent cough were classified as grade 1, those with a moderate cough were classified as grade 2, and those with a severe cough were assigned a grade of 3 [20]. Hoarseness was rated from 0 (none) to 3 (unable to produce a sound), with 1 indicating mild (noted by the patient) and 2 moderate (obvious to the observer) hoarseness [21]. In terms of dysphagia, grade 0 represented no dysphagia, grade 1 mild dysphagia, grade 2 moderate dysphagia (no or occasional difficulty in swallowing liquid; difficulty swallowing solids occasionally or in particular foods), and grade 3 indicated severe dysphagia (occasional or no difficulty in swallowing liquid; frequent difficulty in swallowing or in swallowing most solid foods) [22]. Any complication rated at ≥ 2 was considered significant.

### Sample size and statistical analysis

The pre-test data indicated that 70% of group C patients, 50% of group G patients, and 30% of group A patients had POST within 48 h after surgery. PASS15.0 was used to calculate the sample size, and α = 0.05 and β = 0.2, respectively, were the significance thresholds of the hypothesis test. It was predicated on the assumption that 20% and 40% decreases in POST incidence in groups G and A, respectively, compared to group C, were statistically significant. This showed that the required number of patients was 91. Allowing for a dropout rate of 20%, a total of 114 patients were required, with an average of 38 patients in each group.

Continuous data are presented as mean (standard deviation) or median (25th-75th percentiles), according to the normality of the data distribution. Analysis of variance (ANOVA) was used for comparing normally distributed data while the Kruskal-Wallis test was used to compare non-normally distributed data. Categorical data, including the rates of postoperative complications, are presented as frequencies (percentages). Statistical significance across groups was assessed using either the Chi-square test or the Fisher exact test. The reported p-values underwent Bonferroni corrections. P-values < 0.05 were considered statistically significant. All data were analyzed using SPSS 25.0 for Windows (IBM Corp., Armonk, NY, USA).

## Results

**Fig. 2:**
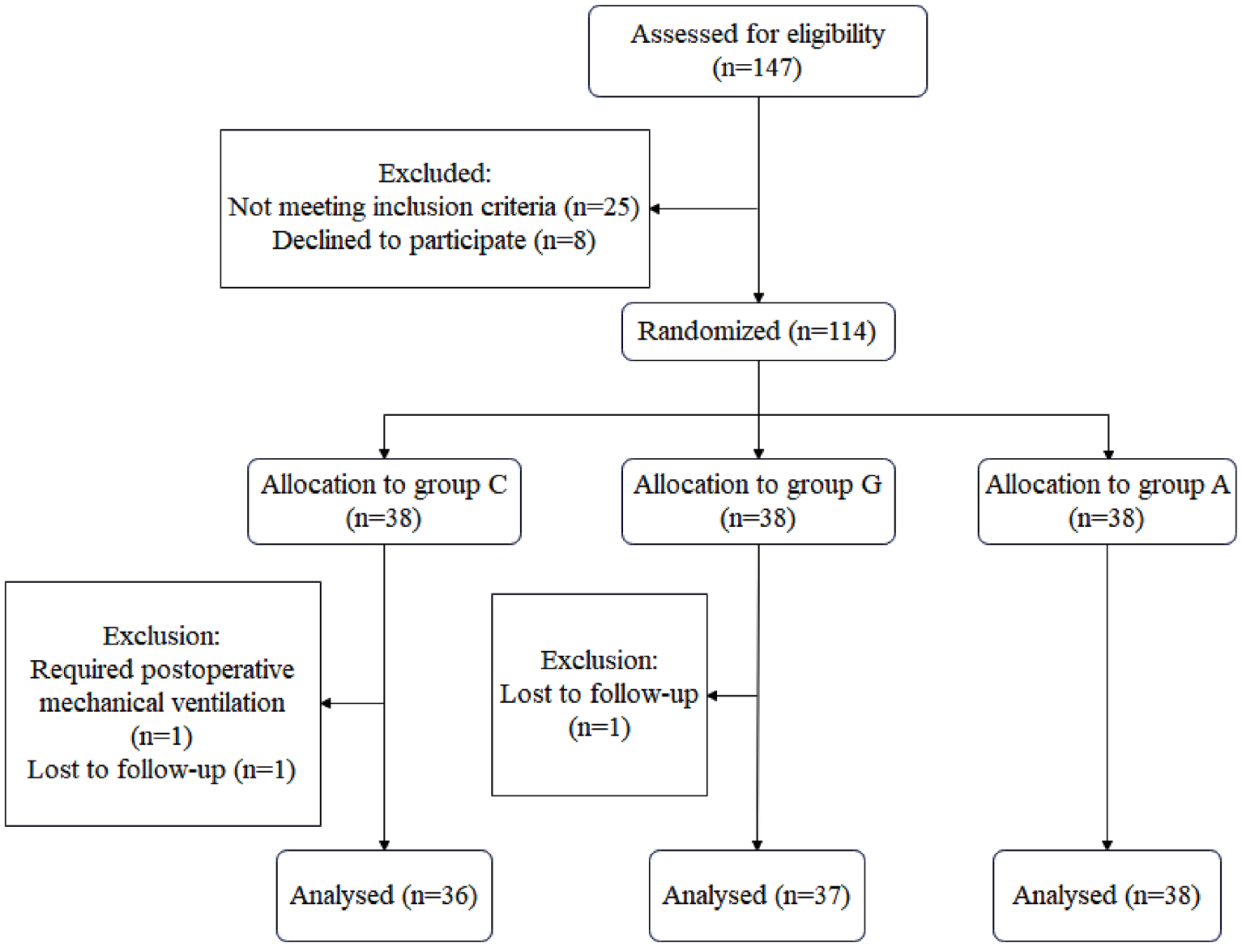
CONSORT flowchart. Patients were randomly assigned to three groups (groups C, G and A) to receive different methods of ETT cuff pressure monitoring, following a computer-generated randomization code

The participants were recruited from March to June 2023. Only 114 of 147 potentially eligible patients were included, and 3 patients (one who needed postoperative mechanical breathing and two who were lost to follow-up) were excluded from the analysis (Fig. 2). Age, sex, body mass index, ASA physical classification, Mallampati classification, surgery type, and surgical position were similarly distributed throughout the three groups (Table 1). Furthermore, there were no significant variations observed in the size of the endotracheal tube, volume of cuff inflation during intubation, duration of surgery, duration of anesthesia, and duration of intubation among the different groups (Table 1).

**Table 1:** Patients and operation characteristics

	Group C (*n* = 36)	Group G (*n* = 37)	Group A (*n* = 38)
Age (yr)	49.5(39.8–56.5)	55.0(50.5–60.0)	51.5(43.5–59.0)
Female, n(%)	20(55.6)	19(51.4)	18(47.4)
BMI (Kg m^− 2^)	24.8(2.7)	24.8(2.5)	24.5(3.2)
ASA classification, n(%)			
I	0(0.0)	2(5.4)	0(0.0)
II	35(97.2)	32(86.5)	35(92.1)
III	1(2.8)	3(8.1)	3(7.9)
Mallampati classification, n(%)			
I	14(38.9)	12(32.4)	14(36.8)
II	22(61.1)	25(67.6)	24(63.2)
Operation duration (min)	142.5(120.0-197.5)	150.0(115.0-172.5)	140.0(108.8–175.0)
Intubation duration (min)	208.6(63.9)	202.4(58.4)	195.8(60.9)
Anaesthesia duration (min)	170.0(140.0-218.8)	165.0(140.0-197.5)	167.0(127.8-202.5)
Cuff inflation volume (ml)	4.0(4.0–5.0)	4.0(4.0–5.0)	4.0(4.0–5.0)
Initial cuff pressure (cmH_2_O)		40.0(29.0–50.0)	38.0(32.0-43.3)
Tube size, n(%)			
7.0	20(55.6)	18(48.6)	18(47.4)
7.5	16(44.4)	18(48.6)	19(50.0)
8.0	0(0.0)	1(2.7)	1(2.6)
Type of Surgery, n(%)			
Spinal	16(44.4)	17(45.9)	16(42.1)
Gynecological	7(19.4)	6(16.2)	7(18.4)
Neurosurgical	6(16.7)	7(18.9)	5(13.2)
Urological	2(5.6)	2(5.4)	2(5.3)
Others	5(13.9)	5(13.5)	8(21.1)
Surgical position, n(%)			
Supine	15(41.7)	17(45.9)	19(50.0)
Prone	15(41.7)	13(35.1)	13(34.2)
lithotomy	3(8.3)	5(13.5)	2(5.3)
Lateral	3(8.3)	2(5.4)	4(10.5)

Abbreviations: BMI, Body Mass Index; ASA, American Society of Anesthesiologists. SD, standard deviation. The values are expressed as mean(SD), median (25-75th percentiles), or number of patients (percentage). *P* < 0.05 is considered statistic significant

### Incidence of airway symptoms

**Fig. 3:**
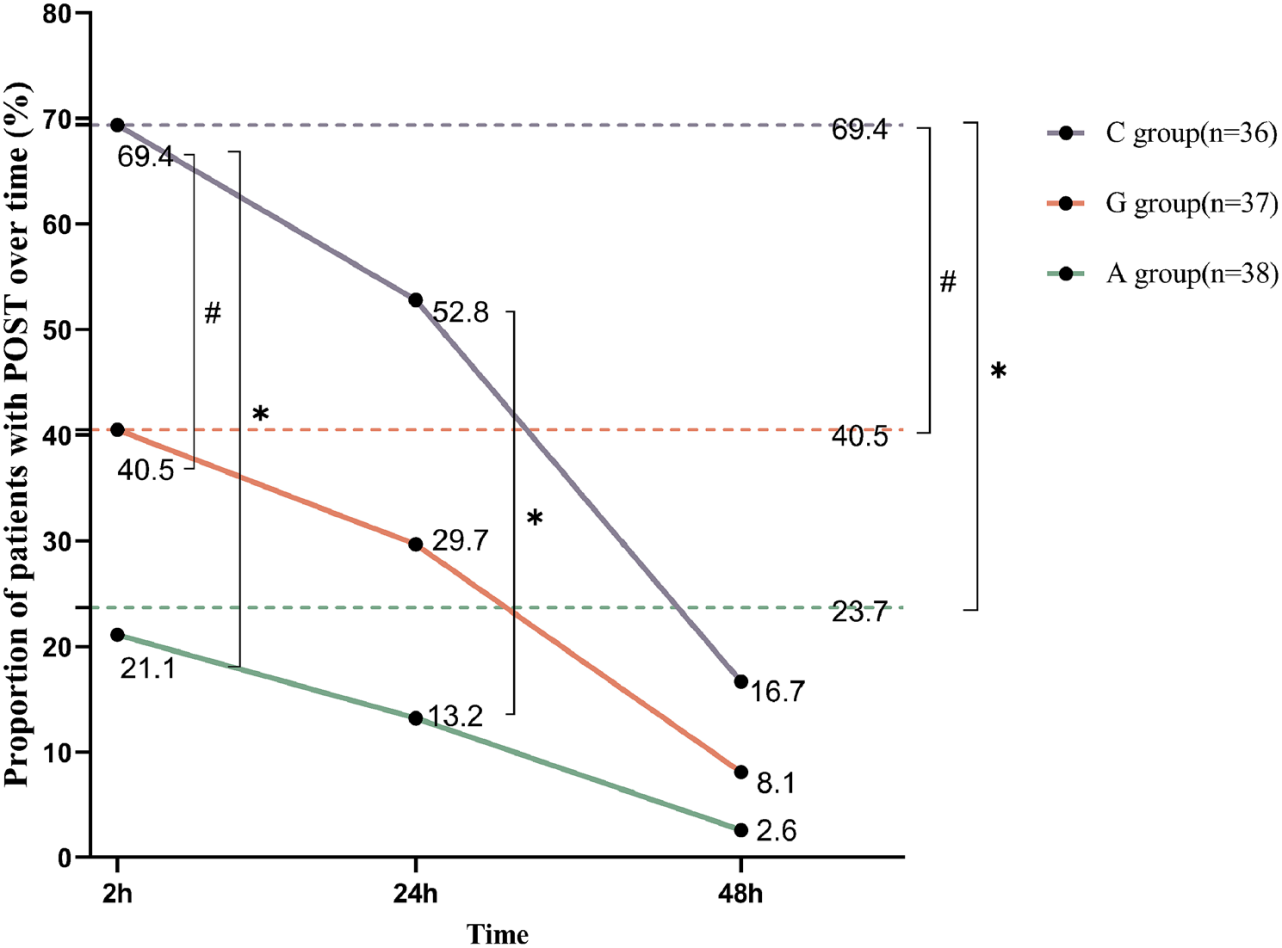
The proportion of patients with POST over time. * represents *P* < 0.05 when comparing groups A and C. # represents *P* < 0.05 when comparing groups G and C. %represents *P* < 0.05 when comparing groups A and G. All the P-values underwent Bonferroni corrections. The dotted line in the background represents the proportion of patients experiencing at least onece POST within 48 h after surgery. POST, postoperative sore throat

During the 48 h after surgery, 69.4% of patients in group C experienced POST; this was significantly higher than the patients in groups G and A (40.5% and 23.7%, *p* = 0.013 and *p* < 0.001, respectively, Fig. 3). At 2 h after the operation, the incidence of POST in group C (69.4%) was significantly higher than that in groups G (40.5%) and A (21.1%) (*p* = 0.013 and *p* < 0.001, respectively, Fig. 3), while at 24 h, the incidence of POST in group A was significantly lower than that in groups C and G (*p* = 0.001 among groups, Fig. 3). At 48 h postoperatively, the difference in the incidence of POST among the three groups was not statistically significant (Fig. 3). There was no significant variation in the occurrence of hoarseness, cough, and dysphagia across the groups at any time point (Table 2).

### Incidence of significant (grade ≥ 2) airway symptoms

The proportion of patients who experienced at least one episode of significant airway complications during the 48 h after surgery was determined. In group A, 10.5% of patients experienced at least one significant episode of POST, which was less than that observed for groups C (41.7%, *p* = 0.002) and G (24.3%, *p* = 0.115) (*p* = 0.009 among groups). However, there was no statistically significant difference in the incidence of significant POST between groups G and C. Simlarly, in group A, 26.3% of patients experienced significant hoarseness at least once, which was also less than that seen in groups C (58.3%, *p* = 0.005) and G (40.5%, *p* = 0.191) (*p* = 0.02 among groups). None of the patients experienced significant coughing, and only one patient in group G suffered significant dysphagia. Details of the number of patients experiencing significant complications are provided in the supplemental materials.

**Table 2:** Incidence of Hoarseness, cough and dysphagia

Outcome	Group C (*n* = 36)		Group G (*n* = 37)	Group A (*n* = 38)	P values
Hoarseness, n(%)					
2 h		24(66.7)	20(54.1)	15(39.5)	0.064
24 h		19(52.8)	17(45.9)	12(31.6)	0.169
48 h		6(16.7)	2(5.4)	2(5.3)	0.171
overall		24(66.7)	20(54.1)	15(39.5)	0.064
Cough, n(%)					
2 h		4(11.1)	3(8.1)	3(7.9)	0.843
24 h		4(11.1)	2(5.4)	5(13.2)	0.532
48 h		0(0.0)	0(0.0)	2(5.3)	0.327
overall		7(19.4)	4(10.8)	8(21.1)	0.452
Dysphagia, n(%)					
24 h		2(5.6)	2(5.4)	3(7.9)	1.00
48 h		0(0.0)	2(5.4)	0(0.0)	0.212
overall		2(5.6)	2(5.4)	3(7.9)	1.00

The values are expressed as number of patients (percentage). *P* < 0.05 is considered statistic significant. The overall represents the incidence of experiencing at least once symptoms within 48 h postoperatively

Group C had considerably worse POST than group A at both 2 and 24 h postoperatively, with scores of 1 (0–2) and 1 (0–1) (compared to 0 (0–0), *p* < 0.001 for 2 h and *p* = 0.001 for 24 h). POST in group G at 2 h postoperatively was graded as 0 (0-1.5) which was milder than group C (P = 0.024). At 2 h postoperative, the severity of hoarseness was rated as 0 in group A (0–2) and 2 (0–2) in group C (*p* = 0.006). Cough and dysphagia severity did not vary significantly across the groups. Details of the severity of all airway problems are provided in the supplementary materials.

## Discussion

According to the results of the present study, the use of an automated cuff controller or cuff pressure gauge substantially decreased the incidence of POST within 48 h, and the group using automated cuff controller had a decreased incidence of significant POST and hoarseness. This outcome essentially confirms our hypothesis.

After intubation, it is crucial to control the pressure within the ETT cuff as part of artificial airway care [23]. Blood flow in the tracheal mucosa begins to decrease when the ETT cuff pressure is more than 30 cmH_2_O, and when the pressure in the cuff approaches 50 cmH_2_O for 15 min, ischemic damage to the tracheal mucosa occurs [24]. Moreover, there is an apparent connection between ETT cuff pressure and the occurrence of postoperative airway symptoms, such as sore throat and hoarseness. According to study conducted by Zhao et al. [25], following intubation while under general anesthesia in Xinjiang, 79.8% of ETT cuff pressure was over 30 cmH_2_O, and 70.9% of patients developed POST. These outcomes align with the results obtained from the control group in our study. Through careful monitoring, the rate of POST decreased to 45.5% and 23.7% in the respective experimental groups.

Cuff pressure gauges and manometers were used extensively as monitoring tools in earlier investigations [11, 12]. Essentially, it is a measurement that relies on applying manual pressure, either through a gauge or a manometer. The incidence of POST was 40.5% in group G in this study, despite regular monitoring of cuff pressure using a pressure gauge every hour during the entire surgical procedure. Our analysis suggests that the intermittent monitoring may have failed to detect the temporary rise in cuff pressure caused by procedural operations or changes in posture. During thyroid surgery, Jung-Hee Ryu et al. [11] employed a manometer to monitor, and 61% of patients developed POST. We consider thyroid surgery as a risk factor for POST, so we chose a broader range of operation types.

POST incidence as a secondary event was 20% and 8% when Jain et al. [26] evaluated two ways of monitoring ETT cuff pressure using a manometer and an automated cuff controller, respectively, in 100 neurosurgery procedures. In contrast, we found that POST monitoring with the gauge and cuff controller had an incidence of 40.5% and 23.7%, respectively. Our study differs in that it compared numerous surgical procedures, including neurosurgery. Furthermore, our controller preserved a pressure range of 25 to 30 cmH_2_O, while the preceding automated cuff controller maintained the pressure constant at 25 cmH_2_O. Moreover, our study measured the incidence of POST at various periods, while earlier studies did not estimate POST at different time points. Using POST and other postoperative airway complications as the primary outcome, our study also established a control group and found that the incidence of POST was markedly reduced to a value of 23.7% when monitored by an automated cuff controller. This was lower than the incidence in the control group and the cuff pressure gauge group. Monsel et al. [27] found that constant regulation of cuff pressure improved pressure stability and decreased variability without compromising cuff tightness. Traditional manual pressure gauge measurement, on the other hand, has a number of limitations, including (1) the cuff pressure cannot be fed back at any time, making it impossible to deflate a cuff that has inflated to an unsafe level, (2) gas in the cuff may be lost during the measurement process itself, and (3) staff compliance with pressure monitoring is low. The cuff pressure gauge and the automated cuff controller did not vary significantly, although a larger sample size might reveal a difference.

The use of direct laryngoscopy, ETT size, intubation length, and intubation skill of the anesthesiologist are all potential risk factors for POST [2]. These were similar in all groups, indicating the reliability of these findings. Furthermore, there was no substantial variation between the groups for the occurrence of hoarseness, cough, and dysphagia in the initial 48 h after the operation. This aligns with earlier study findings [11]. Our study revealed that patients who utilized the automatic cuff controller had significantly reduced hoarseness severity. The application of a high-pressure cuff on the airway wall can potentially damage the recurrent laryngeal nerve. This nerve is responsible for postoperative hoarseness and is located in the groove between the esophagus and the trachea. It was found that the occurrence of dysphagia within 48 h after surgery did not vary significantly between the three groups. A possible reason is the relatively small samples size. However, ensuring appropriate cuff pressure of the ETT during surgery is known to prevent dysphagia [28–30].

There are several advantages to this investigation. Currently, the majority of study on the automated cuff controller has focused on individuals undergoing prolonged mechanical ventilation in the intensive care unit [16, 17]. In our investigation, the automated cuff controller was applied routinely and found to markedly reduce the incidence of several postoperative complications, including sore throat, and is also convenient for anesthesiologists. However, there are some limitations to this study. First, we did not collect data regarding the intraoperative cuff pressure as our cuff controller could only maintain pressure in a fixed range and could not measure the actual value of the cuff pressure. Another limitation is the exclusion of a specific surgical procedure, as our main objective was to investigate the applicability of cuff pressure monitoring across various surgical interventions. Indeed, specific surgeries like thyroid or cervical spine surgeries may be high-risk of POST development [31–33], which should be further investigated in the future.

In summary, this study showed that in patients receiving general anesthesia for tracheal intubation, using the cuff pressure gauge or an automated cuff controller can effectively decrease the occurrence of POST. Anesthesiologists should prioritize patient monitoring and effectively regulate intraoperative cuff pressure.

### Electronic supplementary material

Below is the link to the electronic supplementary material.

Supplementary Material 1

## Data Availability

The datasets used and/or analyzed during the current study are available from the corresponding author upon reasonable request.

## Abbreviations

POST: postoperative sore throat
ETT: endotracheal tube
PACU: post-anesthesia care unit
