# Supplementary material for: The effect of different endotracheal tube cuff pressure monitoring systems on postoperative sore throat in patients undergoing tracheal intubation: a randomized clinical trial
Source: BMC Anesthesiol. 2024 Mar 25;24:115. doi: 10.1186/s12871-024-02499-5 (PMC10962134; doi:10.1186/s12871-024-02499-5)
Supplement: Supplementary file 1 — Supplementary Material 1 [file 12871_2024_2499_MOESM1_ESM.docx]

**Supplemental material**

| Table 1. Proportion of patients with significant (grade ≥2) POST, hoarseness,cough and dysphagia. | | | | |
| --- | --- | --- | --- | --- |
|  | Group C (n=36) | Group G (n=37) | Group A (n=38) | P values |
| POST (n,%) | 15(41.7) | 9(24.3) | 4(10.5)^#^ | 0.009 |
| Hoarseness (n,%) | 21(58.3) | 15(40.5) | 10(26.3)^#^ | 0.02 |
| Cough (n,%) | 0(0.0) | 0(0.0) | 0(0.0) |  |
| Dysphagia (n,%) | 0(0.0) | 1(2.7) | 0(0.0) |  |
| Abbreviations: POST,postoperative sore throat. The values are expressed as number of patients (percentage). ^#^Bonferroni-corrected *P*<0.05 vs group C. P < 0.05 is considered statistic significant. | | | | |

| Table 2. Severity of POST, Hoarseness,Cough and Dysphagia. | | | | | |
| --- | --- | --- | --- | --- | --- |
|  | Group C (n=36) | | Group G (n=37) | Group A (n=38) | P values |
| POST | | | | | |
| 2h | | 1(0-2) | 0(0-1.5)^#^ | 0(0-0)^#^ | ＜0.001 |
| 24h | | 1(0-1) | 0(0-1) | 0(0-0)^#^ | 0.001 |
| 48h | | 0(0-0) | 0(0-0) | 0(0-0) | 0.179 |
| Hoarseness | | | | | |
| 2h | | 2(0-2) | 1(0-2) | 0(0-2)^#^ | 0.008 |
| 24h | | 1(0-2) | 0(0-1) | 0(0-1) | 0.062 |
| 48h | | 0(0-0) | 0(0-0) | 0(0-0) | 0.144 |
| Cough | |  |  |  |  |
| 2h | | 0(0-0) | 0(0-0) | 0(0-0) | 0.867 |
| 24h | | 0(0-0) | 0(0-0) | 0(0-0) | 0.513 |
| 48h | | 0(0-0) | 0(0-0) | 0(0-0) | 0.144 |
| Dysphagia | |  |  |  |  |
| 24h | | 0(0-0) | 0(0-0) | 0(0-0) | 0.894 |
| 48h | | 0(0-0) | 0(0-0) | 0(0-0) | 0.133 |
| Abbreviations: POST,postoperative sore throat. SD, standard deviation. The values are expressed as median (25-75^th^percentiles). ^#^Bonferroni-corrected *P*<0.05 vs group C. P < 0.05 is considered statistic significant. | | | | | |
